# Quantitatively Characterizing the Ligand Binding Mechanisms of Choline Binding Protein Using Markov State Model Analysis

**DOI:** 10.1371/journal.pcbi.1003767

**Published:** 2014-08-07

**Authors:** Shuo Gu, Daniel-Adriano Silva, Luming Meng, Alexander Yue, Xuhui Huang

**Affiliations:** 1 Department of Chemistry, Institute for Advance Study and School of Science, The Hong Kong University of Science and Technology, Clear Water Bay, Kowloon, Hong Kong; 2 Department of Biochemistry, University of Washington, Seattle, Washington, United States of America; 3 Division of Biomedical Engineering, Institute for Advance Study and School of Science, The Hong Kong University of Science and Technology, Clear Water Bay, Kowloon, Hong Kong; 4 Center of Systems Biology and Human Health, Institute for Advance Study and School of Science, The Hong Kong University of Science and Technology, Clear Water Bay, Kowloon, Hong Kong; Max Planck Institute of Colloids and Interfaces, Germany

## Abstract

Protein-ligand recognition plays key roles in many biological processes. One of the most fascinating questions about protein-ligand recognition is to understand its underlying mechanism, which often results from a combination of induced fit and conformational selection. In this study, we have developed a three-pronged approach of Markov State Models, Molecular Dynamics simulations, and flux analysis to determine the contribution of each model. Using this approach, we have quantified the recognition mechanism of the choline binding protein (ChoX) to be ∼90% conformational selection dominant under experimental conditions. This is achieved by recovering all the necessary parameters for the flux analysis in combination with available experimental data. Our results also suggest that ChoX has several metastable conformational states, of which an apo-closed state is dominant, consistent with previous experimental findings. Our methodology holds great potential to be widely applied to understand recognition mechanisms underlining many fundamental biological processes.

## Author Summary

Molecular recognition plays important roles in numerous biological processes including gene regulation, cell signaling and enzymatic activity. It has been suggested that molecular recognition employs a variety of mechanisms, ranging from induced fit to conformational selection. In many realistic systems, conformational selection and induced fit are not mutually exclusive. An analytical flux analysis has been developed to determine the contribution of each model, but it is extremely challenging to obtain the necessary kinetic parameters for this flux analysis through experimental techniques. In this work, we have developed an approach integrating Markov State Models, molecular dynamics simulations, and flux analysis to tackle this problem. Using this approach, we have quantified the recognition mechanism of the choline binding protein to be ∼90% conformational selection dominant in the experimental conditions. Our methodology provides a way to quantify the molecular recognition mechanisms that are extremely difficult to be directly accessed by experiments. This opens up numerous possibilities for *in silico* design to fine tune the recognition event either to increase the degree of conformational selection or induced fit, so that new properties could be created to accommodate the needs of protein engineering, drug development and beyond.

## Introduction

Protein-ligand recognition plays a key role in many aspects of biological processes, such as enzyme catalysis, substrate translocation, and drug therapy [1]. Current studies indicate two prevailing models to address the recognition process: the induced fit model (where ligand binding induces conformational changes of the protein) and the conformational selection model (where the ligand selects a pre-existing conformation of the protein to bind), both of which describe extreme situations (SI Fig. S1) [2]–[10]. Recent studies, however, suggest that many realistic systems show characteristics of both mechanisms [11]–[17].

A better understanding of the role of the two models may lead to an increased utilization of protein engineering techniques – for example we may fine tune their respective contributions to allow the creation of new properties [18]. In particular, augmenting the relative contribution of the induced fit mechanism might increase the binding specificity of a protein receptor. Direct applications of such protein engineering can also lead to better chemical sensors [19].

Hammes *et al.* have developed an analytical model based on flux analysis to determine the contribution of conformational selection and induced fit mechanism [20]. However, difficulties arise in obtaining the thermodynamic and kinetic parameters necessary for the flux analysis from experiments. For example, it is difficult for experiments to directly examine which conformation ligands choose to bind in the conformational selection model, or observe protein conformational changes upon the ligand binding in the induced fit model. Recent progress of NMR techniques such as paramagnetic relaxation enhancement and residual dipolar coupling enable the detection of metastable conformations of the apo protein in solution, and further provide dynamic information for transitions between these conformations [21]–[24]. However, it is still difficult to apply these techniques to monitor the dynamics of the ligand binding process. In any case, as long as one obtains necessary kinetic and thermodynamic parameters, the flux through each pathway can be quantified, allowing one to assign a percentage to the involvement of induced fit or conformational selection for a particular recognition process.

Quantifying the flux is difficult by direct Molecular Dynamics (MD) simulations as well. The current timescale of MD simulations, mostly on the order of tens to hundreds of nanoseconds, is far too short to witness many biological events which occur on the order of milliseconds to seconds [25]. Only if a specific protein-ligand recognition process occurs very quickly can direct MD simulations be efficient, as our previous study on the binding mechanism of L-Lysine-, L-Arginine-, L-Ornithine-binding protein (LAOBP) demonstrated [26]. For that particular system, we used a total of 13 µs MD simulations and Markov State Models (MSMs) to examine the binding events between arginine and LAOBP that occurs at a couple of microseconds.

In this work, we propose a novel approach to qualify the flux following conformational selection and induced fit model for a particular molecular recognition process. By combining the techniques of MSMs for apo protein dynamics, direct MD simulations of the protein-ligand binding and flux analysis, we offer a systematic method for finding the necessary kinetic parameters to quantitatively measure the portion of each flux through two binding pathways. Our application of such an approach in choline binding protein (ChoX) [27], a periplasmic binding protein (PBP) [28] from the ATP-binding cassette transporter ChoVWX, demonstrates that our method can explore the free energy landscape and successfully quantify the independent contribution of two concurrent binding mechanisms – induced fit and conformational selection – in a complex realistic protein-ligand system.

**Figure 1:**
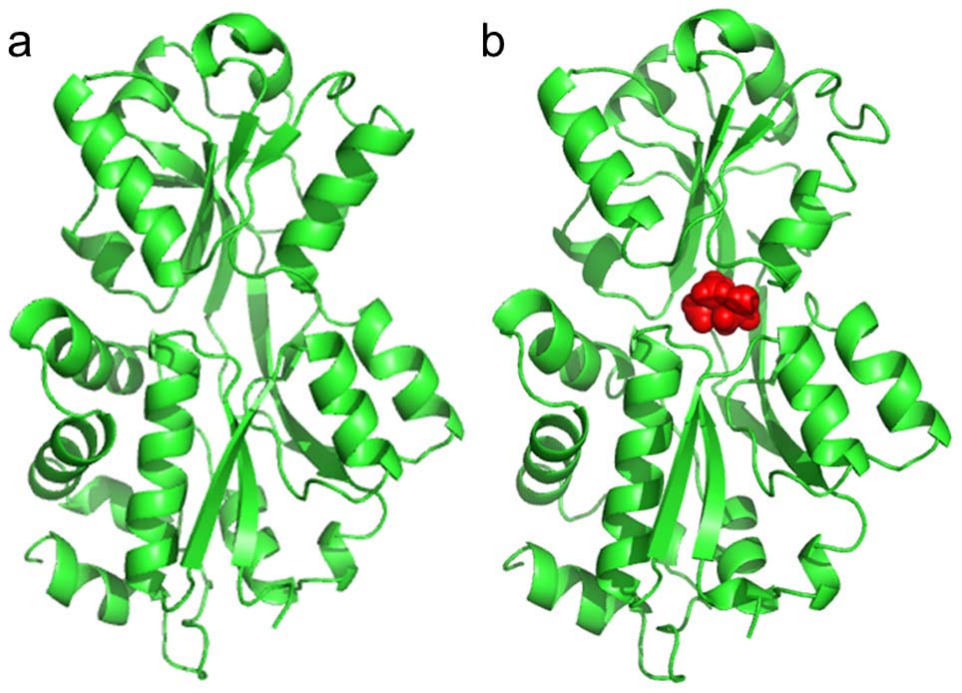
A cartoon representation of the choline-binding protein ChoX in (a) apo-closed (PDB ID: 2RF1) and (b) holo-closed (PDB ID: 2REG) states. The ligand choline is shown in red spheres.

As the periplasmic component of the choline import system, ChoX binds choline or acetylcholine before transferring it to the transmembrane domain of ChoVWX. Currently, X-ray crystallography has characterized several structures of this protein, all of which are in the closed or semi-closed conformations, whether there is a ligand bound or not (Fig. 1) [27], [29]. In comparison to other PBPs, this unique property of ChoX – that it can apparently stay at its closed state without the help of the ligand – made the researchers raise the hypothesis that the binding mechanism of ChoX and its ligand follows the conformational selection model [30].

MSMs are a powerful approach to automatically identify metastable states from short MD simulations and calculate the equilibrium thermodynamic and kinetic properties [31]–[49]. It divides the protein conformational space into a number of non-overlapping metastable states such that the transitions within each state are fast but transitions across different states are slow. Time is coarse grained (with the smallest unit of *τ*, termed as the lag time) to ensure the model is Markovian so that the probability of transitioning from state *i* to *j* only depends on *i* but not any previously visited states. With the help of MSMs, one can extract long time dynamics from short simulations and directly obtain many useful parameters of thermodynamics and kinetics [50]–[60], which can be further utilized in the flux analysis. For example, Noé *et al.* have performed the first flux analysis based on the transition path theory and MSMs to investigate the major pathways for the folding of the WW domain [55]. More recently, Noé and coworkers have also used MSMs to study the mechanisms of protein-ligand association where protein does not undergo substantial conformational changes [61].

The flux analysis proposed by Hammes *et al.* [20] is an useful tool to calculate and compare flux in both induced fit and conformational selection pathways, allowing one to analyze the contribution of these two limiting mechanisms in a complicated binding event. To conduct the flux-based approach, many kinetic parameters are required – namely, the binding constants and transition rates between different metastable conformational states of the system.

The combination of MSMs and MD simulation allows us to obtain such parameters, which are difficult to be directly measured from experimental assays [20]. In our study, the three-pronged approach of MSMs, MD simulation, and flux analysis was successfully applied in ChoX binding event to quantify both limiting recognition mechanisms.

## Results/Discussion

### Construction and validation of MSMs for apo ChoX

We used MD simulations to investigate the free energy landscape of apo ChoX. In particular, we have generated initial twenty 100-ns simulations, ten from apo-closed (PDB ID: 2RF1) and ten from apo-semiclosed (PDB ID: 2REJ) crystal structures [29]; and one hundred 50-ns additional simulations, seeded from random conformations of the previous twenty trajectories. In total, we collected 7 µs of apo simulations and constructed a MSM using the MSMBuilder package [33] (see Methods for the details of model construction). The implied timescale plots flatten at ∼15 ns, indicating that the model is Markovian with this or longer lag time (SI Fig. S2) [33]. We thus selected 20 ns as the lag time to construct our MSM. Since the macrostate-MSM underestimates the kinetics, we computed all the quantitative properties reported in this work such as equilibrium state populations and other kinetic properties based on the 500-state microstate-MSM. To better visualize the conformational dynamics of ChoX, we have also lumped the 500 microstates into 5 metastable states, denoted as S1 to S5 with descending populations (see the Methods section for details).

### Free energy landscape of apo ChoX

**Figure 2:**
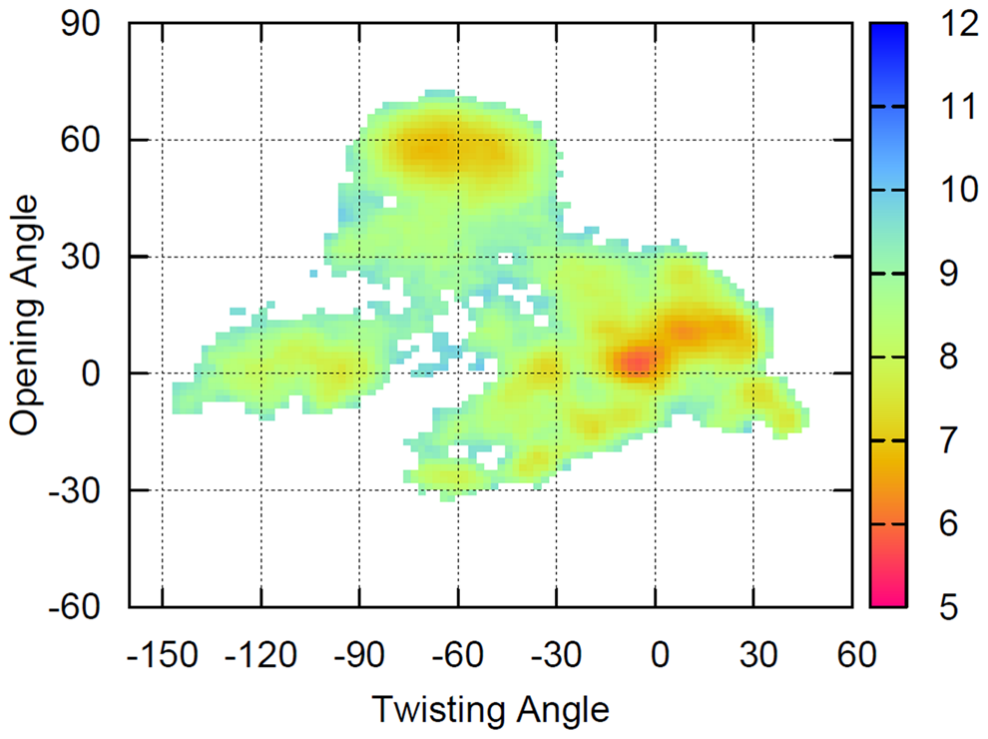
Projections of the free energy landscape onto the opening and twisting angles for the apo ChoX. The free energy profiles were obtained by averaging over contributions from five different metastable states of MSMs weighted by their equilibrium populations. The unit of the color bar is kT. See SI Fig. S9 and Methods for the definition of opening and twisting angles.

The projection of the free energy landscape of apo ChoX on the domain-domain opening and twisting angle is plotted in Fig. 2. It is clear that the most populated region is near (0°, 0°), corresponding to an apo-closed crystal structure. Our simulations demonstrate that such a conformation lies in the most dominant metastable state S1 with a population of ∼47%. This result is consistent with previous X-ray crystallography research, which noted that ChoX could exist in the closed conformation without the help of a ligand [29]. In addition to this metastable state, we found other states with different degrees of opening or twisting. Such metastable conformations were not discovered by X-ray crystallography, possibly due to the very compact crystal environment and strong contacts between unit cells present in the available apo structures of ChoX (SI Fig. S3) [29].

We also studied kinetics for the transitions between these metastable states. The mean first passage times (MFPTs) were calculated for each pair of states (SI Table S1), and these timescales are on the order of microseconds.

### Structural and dynamic features of metastable states of apo ChoX

**Figure 3:**
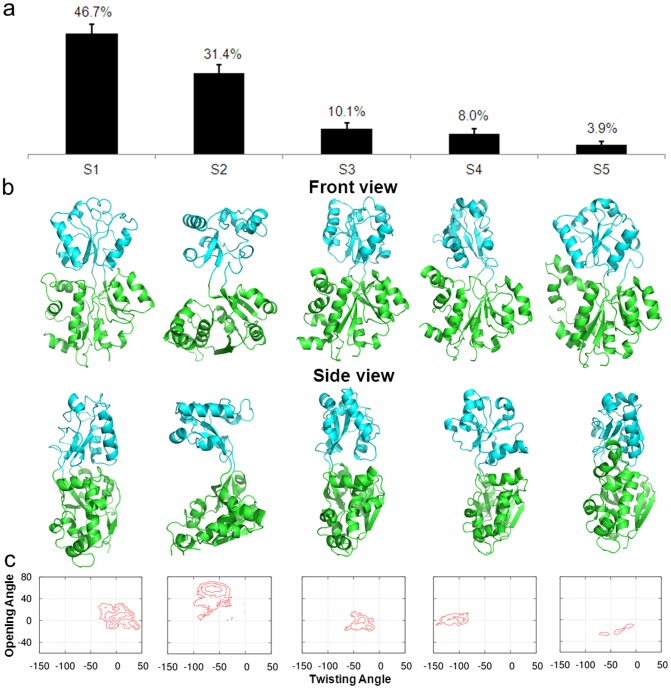
(a) The equilibrium populations of five metastable states obtained from the MSM for apo ChoX. (b) Representative conformations from these five states. The two domains of the protein are colored in cyan and green respectively, and two viewpoints, front and side views, are shown. (c) Projections of free energy landscape on the protein opening and twisting angle for each metastable conformational state. The interval between two adjacent contour levels is 1 kT.

The structural features of the five metastable states are displayed in Fig. 3. The most populated state S1 is a closed conformation very similar to those discovered crystal structures. S2 is an open-and-twisted conformation with an essential hydrogen bond between N229 and G232 at the back of the hinge region. S3 is a closed conformation with a small opening at the side of the domain-domain interface, which is large enough to allow the diffusion of the ligand to the binding site (Fig. 3b). S4 is twisted to a very large degree. S5 is another closed structure similar to S1 with a different orientation of the helix containing residues 262–275. Further investigations show that hydrogen bonds may play an important role to stabilize these metastable conformations. One example from the metastable state S2 involves N229 and G232. When N229 was mutated to alanine or G232 was mutated to bulkier tyrosine to diminish the hydrogen bonds, fast transitions (within 50-ns) were observed from S2 to S1 (SI Fig. S4) compared to the wild type (∼2.07 µs), demonstrating the critical contribution of the hydrogen bond (N229-G232) to the metastability of S2.

### Ligands can selectively bind to the closed ChoX conformation to reach the bound states

We performed ten 50-ns simulations for each of the five metastable states by introducing ligands to the system. To increase the chance of observing a binding event within the length scale of MD simulations, we have added 20 ligands to the system (at a concentration of ∼0.069M). In each simulation, 20 ligands were randomly placed in the simulation box away from the binding site with the minimum distance of 17 Å and the protein conformations were randomly chosen from each metastable state.

For S1 with closed protein conformations (S1+L), we discovered two out of ten simulations where the ligand recognized the target and bound to it. In order to enhance the sampling, we have performed additional twenty 50-ns MD simulations and three of them were identified with binding events.

**Figure 4:**
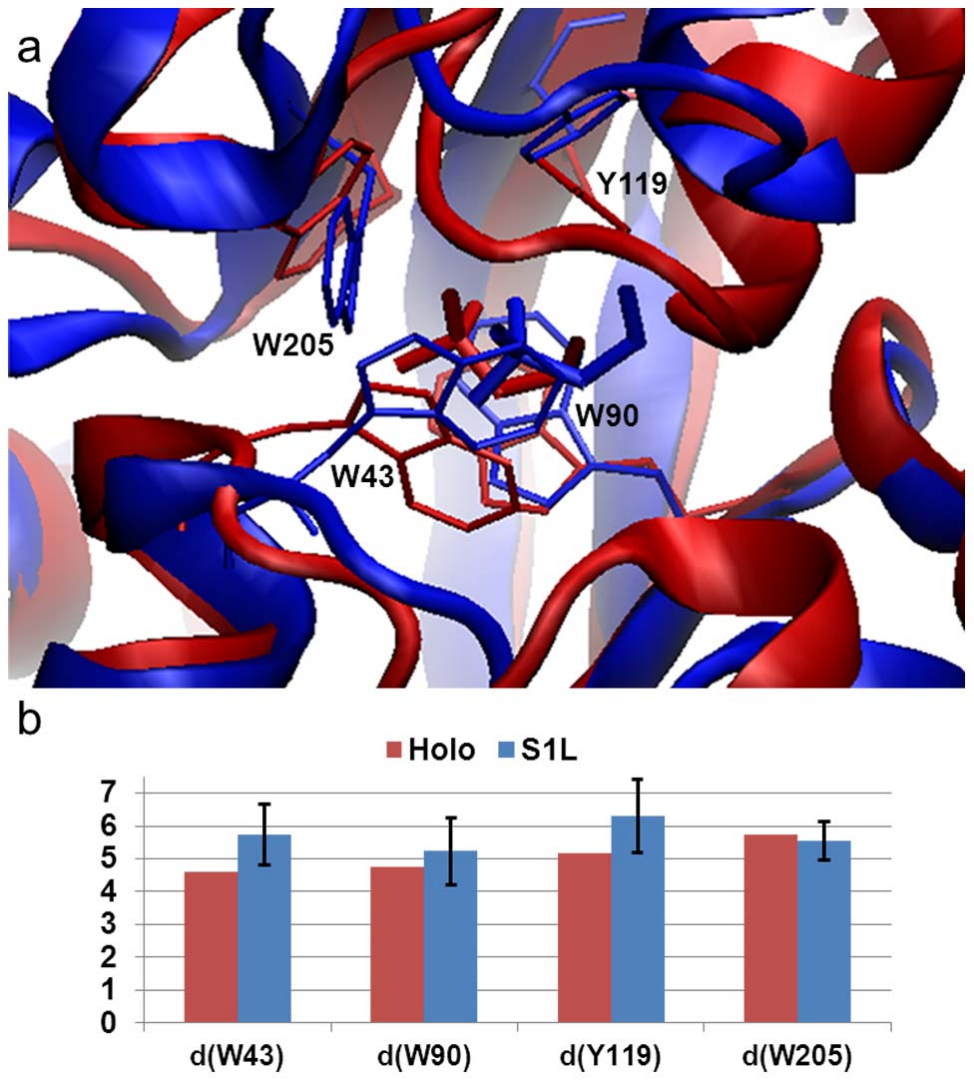
Choline can selectively bind to the closed protein conformation S1 to reach the holo state. (a) The X-ray bound conformation (red, PDB ID: 2REG) is superimposed with a conformation selected from MD simulations in the presence of ligands and with smallest RMSD to the bound state (blue). Four critical residues in the binding sites that are in direct contact with the ligand are highlighted in stick representation. (b) Distances (Å) between the center of mass of ligand and four centers of mass of critical residues in the binding site obtained from the holo structure (red bars) and five MD trajectories in which only those conformations after the ligand binding have been included in the analysis (blue bars).

The pathways of the ligand binding to S1 can be mainly characterized as conformational selection, and these binding simulations achieved similar conformation compared to the X-ray bound structure with a RMSD as small as 1.6 Å of protein Cα atoms which are within 8 Å to the binding site (Fig. 4a). In addition, we examined the distances of the ligand choline to four essential residues at the binding site after the ligand binds to S1, and the values are similar to those from crystal structure. These results indicate that MD simulations have the capability to predict the bound state *in silico*.

### The ligand binding can also induce the conformational changes of the metastable state S3 to reach the bound state

**Figure 5:**
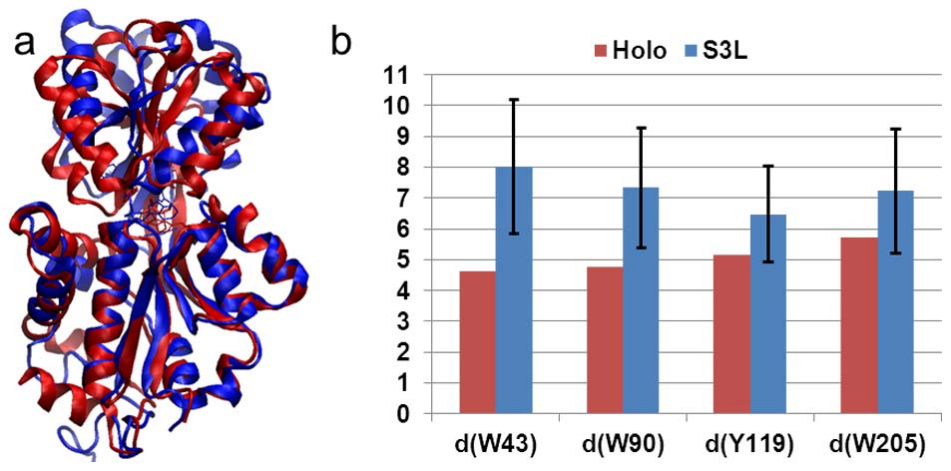
MD simulations initiated from the state S3 that exhibit spontaneous ligand binding. (a) A representative conformation of S3L (blue) is overlaid with the X-ray bound state (red, PDB ID: 2REG). (b) The same as Fig. 4b except that the distances computed from 7 50-ns ligand binding MD simulations that the ligand binds state S3.

In addition to these ligands that can directly bind to the closed conformation S1, we also found in other simulations that the ligand can interact with the conformation from the state S3 and, at the order of tens of nanoseconds induce the conformational change to the bound conformation S1L. Recall S3 was a closed and twisted state with a side-opening cavity ready for a ligand to insert. We also demonstrated that, from a distance analysis and an overlay of S3L with the holo crystal structure, the ligand stays close to Y119 and W205 (Fig. 5b). One trajectory was discovered with a transition from S3L to S1L, which suggests the possibility that the ligand can bind ChoX through an induced fit mechanism. Since only a single transition event was observed among ten simulations of S3+L, we have performed additional twenty 50-ns MD simulations of S3L complex to enhance the sampling. Among these twenty simulations, two of them displayed the transitions from S3L to S1L (SI Fig. S5).

For the remaining metastable states S2, S4 and S5, no ligands were found to bind to the correct binding site to form the complex: S2L, S4L or S5L (SI Fig. S6). However, in order to examine whether or not these protein-ligand complexes (S2L, S4L or S5L) if exist can induce the transitions to the bound state S1L, we have modeled these complexes using the AutoDock Vina [62], and initiate ten 50-ns MD simulations from each of these docked conformations. As shown in SI Fig. S7, none of these simulations contained any transitions to the bound state (S1L). These results indicate that the direct transitions from S2L, S3L and S5L to the bound state S1L are unlikely to occur.

### Quantifying the binding flux

From the MD simulations discussed above, we can obtain a rough picture of the hybrid mechanism of conformational selection and induced fit for ChoX. However, the great challenge is to quantify the percentage of each mechanism in complicated scenarios. In order to achieve this, we have applied the flux analysis theory [20], where the flux going through each pathway is utilized to quantify binding mechanisms. At first, the conformational selection pathway can be described as (SI Fig. S1):
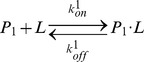
(1)where P_1_ represents the closed conformation S1 of ChoX, and other metastable conformations P_i_ (i = 2–5) can interconvert with P_1_:
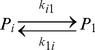
(2)In this work, we consider the conformational selection mechanism in a general context where the ligand selects to bind a certain metastable protein conformation including the ground state (P_1_ in this case).

On the other hand, the induced fit pathway can be described by a two-step process (SI Fig. S1), where the ligand first binds to any metastable conformation P_i_ other than the closed conformation P_1_:
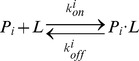
(3)And the binding will further induce the conformational change to reach the bound state:
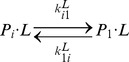
(4)The flux flowing through each of the above two pathways can be derived from the flux analysis theory as the following (see Methods for the details of the derivation):
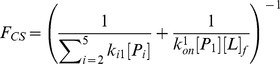
(5)

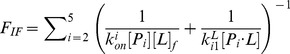
(6)where *F_CS_* and *F_IF_* represent the flux through conformational selection and induced fit pathway respectively. 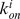 and 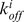 are the kinetic rate constant for ligand binding/unbinding to state i respectively; *k_i1_* is the rate constant for the transition from state i to state 1; 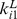 is the rate constant for the transition from the complex S_i_·L to the bound state S_1_·L; and [*L*]*_f_* is the free ligand concentration.

In this study, we have derived important parameters from MSMs and MD simulation that are missing in the flux analysis. Specifically, *k_i1_* can be derived from the transition probability matrix. For MD simulations starting from state S2, S4 and S5, we didn't observe any successful binding events, therefore 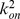, 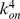, 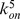 are all set to be zero. For S3, there exist multiple binding and unbinding events in our ten 50-ns MD simulations. Therefore, we have obtained 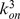 and 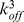 by computing the fraction remaining in the unbound state S3 as a function of time followed by fitting to Eq. 19 (SI Fig. S8). 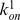 can be derived from MD simulations with ligands in a similar way to 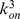 (Eq. 20). At last, we need to derive the values for 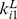
(i = 2–5). Since the simulations of S2L, S4L and S5L didn't show any transitions from each state to S1L (SI Fig. S7), these values (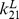, 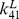, 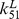) are all set to be zero. For 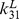, we can obtain its value from twenty S3L MD simulations since the ligand binding further induced the conformational changes to the bound state in a fraction of these trajectories (Eq. 24).

**Figure 6:**
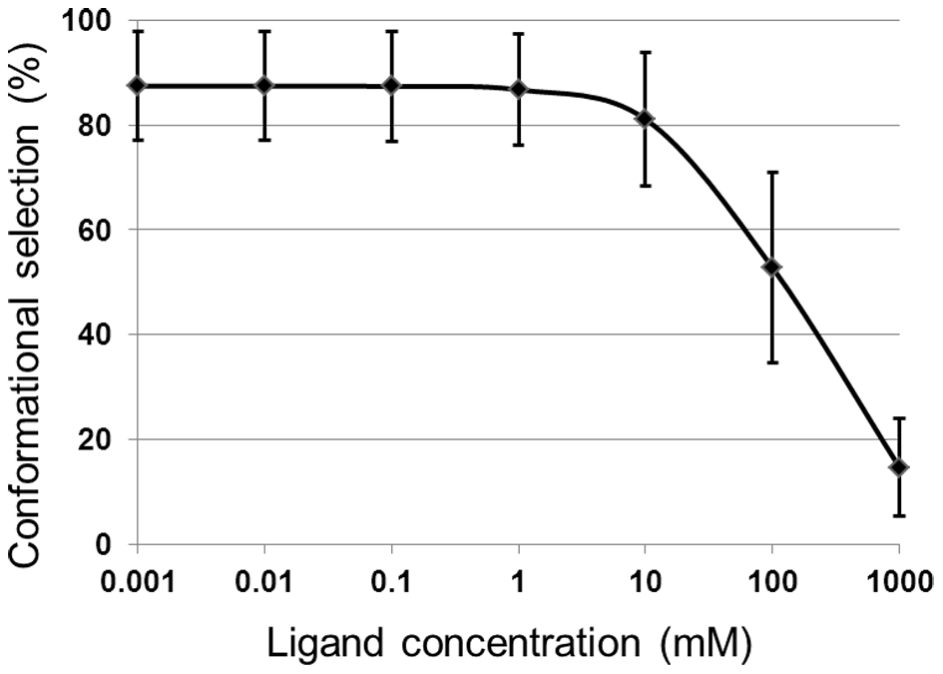
The percentage of conformational selection mechanism as a function of ligand concentration. Conformational selection is dominant for most of the ligand concentration range for ChoX.

We can then proceed to measure the percentage contribution of conformational selection and induced fit mechanism using equations (5) and (6). At the protein concentration fixed to 1 µM, Fig. 6 shows the contribution of conformational selection to the binding pathway depending on the ligand concentration. Conformational selection is dominant for a wide range of the ligand concentration, accounting for around 90% of the binding event at the concentration of choline in the lab conditions (µM scale) [63]. Therefore, we conclude that the binding mechanism of choline to ChoX is dominated by conformational selection.

### Conclusions

In summary, we here propose a novel method that combines MSMs, MD simulations and flux analysis to quantify the binding mechanism of conformational selection and induced fit in complex binding events. In the case study of choline binding to ChoX, we were able to derive all the necessary parameters using MD simulations and MSMs. Based on these parameters, the percentage of each limiting binding mechanism could be quantitatively calculated as a function of ligand concentration. It would be difficult, using common experiments, to obtain these necessary parameters to elucidate these mechanisms. Finally, once the mechanism is quantified, one can further apply other techniques (e.g. *in silico* design) to the biological system to fine tune the binding event either to increase the degree of conformational selection or induced fit, so new properties of macromolecules could be explored and created to accommodate the needs of protein engineering and beyond.

## Methods

### Definition of the opening and twisting angle

We project the free energy landscape of ChoX onto two dihedral angles: the opening and twisting angles. The opening angle is defined as the angle between the normal vectors of the two planes formed by the center of mass of the following groups of Cα atoms (SI Fig. S9a):

Plane A: residues 31–114 & 234–316; 185–194; 159–166.

Plane B: residues 118–230; 185–194; 159–166.

The twisting angle planes are:

Plane C: residues 31–114 & 234–316; 185–194; 46–55.

Plane D: residues 118–230; 185–194; 46–55 [64].

From Principal Component Analysis (PCA) of the apo MD simulations, we found strong correlations between the opening angle and the first eigenvector (R^2^ = 0.77), as well as between the twisting angle and the second eigenvector (R^2^ = 0.53) (SI Fig. S9b). In this work, the degrees of apo-closed and holo-closed crystal structures were shifted to the (0°, 0°) point in corresponding settings.

### MD simulations of the apo ChoX protein

The GROMACS 4.5.4 [65] software and Amber99sb force field [66] were used for all the MD simulations. The procedure is as follows: the protein (and ligand, when present) was solvated in a dodecahedron box with 14, 450 SPC water molecules [67] and enough counter ions to neutralize the system. The system was first minimized with a steepest descent algorithm, followed by a 200 ps MD simulation with position restraints for the heavy atoms. All the simulations were performed at NVT ensemble with 300K of temperature using V-rescale thermostat [68]. The cut-offs for both VDW and short-range electrostatic interactions were set to 10 Å and long-range electrostatic interactions were treated with the Particle-Mesh Ewald method [69]. The time-step was 2 fs and the neighbour list was updated every 10 steps. Water molecules were constrained by the SETTLE algorithm [70] and all protein bonds were constrained by the LINCS algorithm [71].

### Markov state model construction

Using the MSMBuilder package [33], we first applied the k-centre algorithm to cluster all the conformations into 500 microstates based on the Cα atoms of residues in proximity to the binding site (i.e. within 8 Å of the ligand as defined by the holo crystal structure, PDB ID: 2REG). The generated microstates were small, with the average RMSD values to its central conformation in each state of about 1.9 Å. The transition probability matrix (***T***) was obtained by counting the number of transitions observed in the MD trajectories. We then examined the transition probability matrix, and removed two disconnected microstates from our model. The implied timescales (*τ_k_*) obtained from the transition probability matrix ***T*** indicates the aggregated timescales for transitions between groups of microstates.
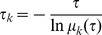
(7)where *μ_k_* is an eigenvalue of the transition matrix with the lag time *τ*.

We have examined the implied timescale plots for this 500-microstate model to select a lag time that ensures the model to be Markovian. As shown in SI Fig. S2, the implied timescale curves plateau at around 15 ns, indicating the model is Markovian with this or longer lag times. Thus we chose a lag time of 20 ns for our final MSM. In order to better visualize the conformational dynamics of apo ChoX, we have further lumped all the microstates into 5 metastable macrostates using the Robust Perron Cluster Analysis (PCCA+) algorithm [72].

The 500-state microstate-MSM was used to compute all the quantitative properties we report in this work. To obtain the populations of metastable states (*P_1_, … P_5_*) from the 500-state microstate-MSM, we simply sum over the equilibrium populations of all the microstates that belong to a certain metastable state: 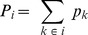. To compute the MFPT between a pair of metastable state *i* and *j* from the microstate-MSM, we first set MFPTs of all the microstates that belong to the destination metastable state *j* to be zero: 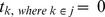. We then computed MFPTs starting from any of the microstate that belong to the metastable state *i:* 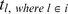. Finally, we obtained the MFPTs from *i* to *j* by performing a weighted average over all the microstates that belong to *i*: 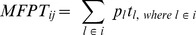.

### Mean first passage time calculation

The MFPT, determined by the following formula, calculates the average transition times between a pair of states [73].
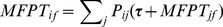
(8)where ***P_ij_*** is the transition probability from state ***i*** to state ***j***, ***τ*** is the lag time of the transition probability matrix ***T***, and ***MFPT_jf_*** is the mean first passage time of the state ***j*** to final state ***f***. The boundary condition is ***MFPT_ff_*** = 0.

### Deriving force field parameters for choline

In order to simulate the process of choline binding to the ChoX protein, we need to obtain the force field parameters for choline. We followed the same procedure as we previously published [74] to derive both bonded and non-bonded force field parameters of choline. Specifically, we have obtained the stretching, bending and torsion parameters by fitting against the Quantum Mechanics (QM) calculations performed using the Density Functional Theory with B3LYP/6-31G* in the Gaussian software [75]. Similar with previous studies [76], we have employed the Restrained ElectroStatic Potential (RESP) method to derive the partial charges from the QM calculations with HF/6-31G*. We have listed all the force field parameters in the format that is compatible with the GROMACS 4.5 software package in SI Text S1, .

### Deriving parameters for flux analysis

The two limiting ligand-binding mechanisms: conformational selection and induced fit can be described by Eq. (1)–(4). Following the flux analysis theory developed by *Hammes et al.* [20], we can then derive the fractional flux passing through each pathway. We note that this flux analysis can be considered as a special case of transition path theory and yields consistent path fluxes for serial and parallel pathways [55], [77]. In particular, if one pathway is consisted of parallel reaction paths, its flux can be written as:
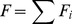
(9)If one pathway contains serial segments, the flux is:
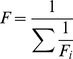
(10)Now, let's consider the conformational selection pathway, which involves two steps. First, different protein metastable conformations (S_i_, i = 2–5) can all interconvert to the closed state (S_1_) at rates k_i1_ (Eq. 2). Therefore, the flux for this segment is: 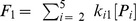, where [P_i_] is the concentration of the state S_i_. Next, the ligand can selectively bind to the closed protein conformation S_1_, to reach the bound state. For this part, the flux is: 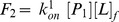 (Eq. 1). Therefore, the flux of conformational selection F_CS_ can be derived as:
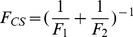
(11)The detailed expression of F_CS_ can be found in Eq. 5.

Following the similar procedure [20], we can also derive the flux for the induced fit pathway. In particular, there exist independent parallel pathways to reach the bound state, where the ligand can first bind to a certain metastable conformational state (S_i_, i = 2–5), and further induced the conformational change to the bound state. The flux of each of these pathways (F_i_) can be written as:
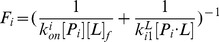
(12)where 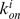 and 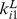 denote to the rate for the ligand binding to state S_i_ (i = 2–5) and the transition rate from the complex S_i_·L to the bound state S_1_·L respectively; 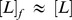 when [L] exceeds. When [L] and [P] are comparable, [*L*]*_f_* is given by Eq. 13:
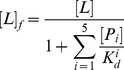
(13)The overall flux for the induced fit pathway (F_IF_) can then be written as:
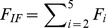
(14)The fractional flux for conformational selection pathway is:
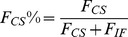
(15)In order to obtain F_CS_%, we need to derive a series of parameters: 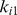
(i = 2–5), 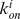
(i = 1–5), 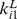
(i = 2–5). 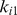 are the transition rate constants from state i to state 1, which can simply be derived from MFPT [58]:
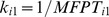
(16)where MFPT_i1_ is the mean first passage time of state i to final state 1. The uncertainties of this set of rates are obtained from bootstrapping the MD dataset (containing N = 138 trajectories) with replacement for N times.

For 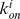, we use our MD simulations with ligand to derive their values. Based on Eq. (1) and (3), we can write the rate equation as:
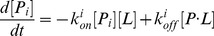
(17)In our simulations, the initial ligand concentration [L]_0_ is twenty times larger than the initial protein concentration [P]_0_: 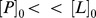, therefore, 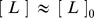. The forward reaction, which is only dependent on k_on_, can then be written as:
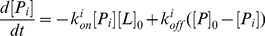
(18)We can solve Eq. 18 to obtain [P_i_]:
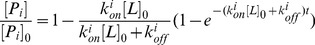
(19)


We can then examine our ligand MD simulations initiated from different protein metastable conformations to obtain 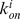 and 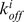. For MD simulations starting from state S2, S4 and S5, we didn't observe any binding events (for distances between the center of mass of ligand and center of mass of four critical residues, W43, W90, Y119, W205 [29], in the binding site to be less than 12 Å), therefore 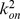, 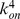, 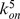 all equal zero (however we estimated their upper limits in Table 1). For S3, there exist multiple binding and unbinding events in our ten 50-ns MD simulations. We have thus computed the fraction remaining in the unbound state (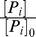) as a function of time, and further fit Eq. 19 to it in order to obtain 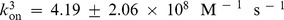 and 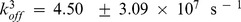 (see SI Fig. S8 for the parameter fitting). We can then obtain 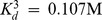. Thus, [P_3_·L] can be calculated by 
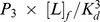
. For S1, we have observed multiple binding events; however none of the unbinding events from our MD simulations, due to the high stability of the bound state. We can then simplify Eq. 18 by only considering the forward reaction and 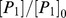 can be written as:
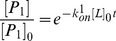
(20)We then obtain 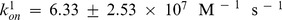.

**Table 1:** Parameters for the flux analysis.

Parameter	Value	Parameter	Value
P_1_	0.467±0.038 µM	k^1^_on_	6.33±2.53×10^7^ M^−1^ s^−1^
P_2_	0.314±0.034 µM	k^2^_on_	<3.06×10^7^ M^−1^ s^−1^
P_3_	0.101±0.022 µM	k^3^_on_	4.19±2.06×10^8^ M^−1^ s^−1^
P_4_	0.080±0.019 µM	k^4^_on_	<3.06×10^7^ M^−1^ s^−1^
P_5_	0.039±0.013 µM	k^5^_on_	<3.06×10^7^ M^−1^ s^−1^
k_21_	5.78±2.29×10^5^ s^−1^	k^L^_21_	<2.11×10^6^ s^−1^
k_31_	7.19±5.40×10^5^ s^−1^	k^L^_31_	2.60±1.60×10^6^ s^−1^
k_41_	1.81±1.00×10^5^ s^−1^	k^L^_41_	<2.11×10^6^ s^−1^
k_51_	2.97±3.09×10^6^ s^−1^	k^L^_51_	<2.11×10^6^ s^−1^
		k^3^_off_	4.50±3.09×10^7^ s^−1^

To further examine the robustness of the definition of the successful binding events, we have compared it with a more specific definition: the heavy atoms of the ligand form contact with atoms belonging to at least three critical residues (among W43, W90, Y119, W205 [29]) in the binding pocket. These two sets of rates only differ slightly (SI Table S2). Furthermore, we have compared the fraction of conformational selection computed based on two different definitions of ligand binding. As shown in SI Fig. S10, the results from two different definitions are also consistent.

In order to examine whether or not the protein undergoes any major conformational changes while collecting data for estimating the binding rates (e.g. 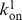, 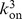, and 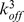), we have projected protein conformations in each binding MD simulation onto a pair of reaction coordinates (opening and twisting angles). As shown in SI Fig. S11, the protein remains in its initial metastable state during the whole course of all the binding MD simulations. These results confirm that our estimations of binding rates are clean.

In order to obtain 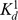, which is the dissociation constant for the ligand directly binding to S_1_, we have constructed a thermodynamic cycle to compute its value (SI Fig. S12):
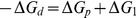
(21)where the free energy 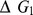 is directly related to 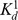 : 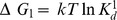.


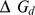
 can be computed from the disassociation constant for the ligand binding 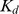 by 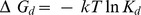, where 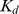 has been obtained experimentally as 2.7 µM [63]. 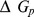 is the free energy difference associated with the conformational transitions from different metastable states to the closed state S_1_:
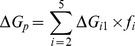
(22)where 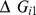 is the free energy difference between S_i_ and S_1_, and f_i_ denotes to the equilibrium population of S_i_:
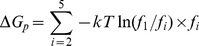
(23)From Eq. 21–23, we can compute the value of 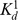 as 4.53 µM.

At last, we need to derive the values for 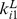
(i = 2–5). As discussed before, there are no binding/unbinding events for S2, S4 and S5, and these values (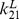, 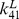, 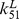) are all set to be zero. For 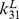, we can obtain the value from the MD simulations initiated from the S3 state with ligand.
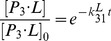
(24)
Eq. 24, helps us to calculate the forward transition rate 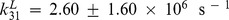.

For the ligand binding/unbinding (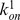, 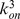 and 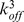) and transition from S3L to S1L (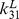). We have performed the bootstrapping analysis to obtain the error bars. In particular, we bootstrapped the MD dataset (containing N MD simulation trajectories with N = 30, 9 and 20 for 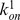, 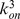/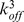, and 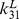 respectively) with replacement for N times.

For rates that are estimated to be zero with no observed transitions (e.g. 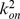, 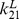), we have estimated the upper limit of these rates by assuming one binding/transition event occurs during our accumulated 500-ns simulation time. We also show that applying these upper limits of the rates to our flux analysis does not change qualitatively the conclusion of this paper: the fraction of the conformation selection slightly decreases from 93% to 82% at experimental concentrations (∼1 µM) as shown in SI Fig. S13.

We have listed all the necessary rate constants with uncertainties/upper limits in Table 1.

## Supporting Information

Figure S1 A schematic diagram representing the conformational selection and induced fit models of protein-ligand recognition.
(TIF)

Figure S2 Implied timescales of microstate and macrostate models. (a). Fifteen slowest implied timescales as a function of lag time computed from the 500-state microstate-MSM. (b). Implied timescales as a function of lag time computed from the 5-state macrostate-MSM.
(TIF)

Figure S3 Crystal contacts experienced by the ChoX in the (a) apo-closed (PDB ID: 2RF1) and (b) apo-semiclosed (PDB ID: 2REJ) X-ray structures. The central unit cell contains two ChoX molecules (in ribbon representations). The surrounding unit cells are shown in surface representations in orange.
(TIF)

Figure S4 MD simulations of two single mutants of apo ChoX, Asn229Ala and Gly232Tyr, that exhibit an accelerated conformational change from the open to the closed state. We have performed three 50-ns MD simulations for each mutant. One of these MD simulations for each mutant is projected onto the opening and twisting angles with a step size of 5-ns. The projections of the apo ChoX free energy landscape (the same as Fig. 3c, and each macrostate is assigned a different color) are also displayed in the same figure.
(TIF)

Figure S5 Protein conformational changes are displayed for three MD simulations where transitions from S3L to S1L occur. The projections of the free energy landscape onto the opening and twisting angles are shown for state S3 (blue) and S1 (red). Each arrow corresponds to a 10-ns segment of the MD simulation. The black cross represents the holo crystal structure. The middle and right panels correspond to the two additional MD simulations containing the transitions from S3L to S1L.
(TIF)

Figure S6 Superimposition of representative snapshots from macrostates S2 (a), S4 (b) and S5 (c) in blue with the X-ray structure of the ChoX bound state (red, PDB ID: 2REG). Each protein conformation is displayed in both front and side views. We did not observe any stable binding events in our MD simulations. A binding event is defined as when distances between the center of mass of the ligand and center of mass of four critical residues (W43, W90, Y119 and W205) in the binding site all to be less than 12 Å. Therefore we consider that the ligands do not bind to these metastable states (S2, S4 and S5).
(TIF)

Figure S7 The projections of protein conformational change on the opening and twisting angles during the course of MD simulations are displayed in black arrowed lines (two trajectories in one panel). The simulations initiated from S2L, S4L, and S5L are plotted in (a), (b), and (c) respectively. The projections of the apo protein free energy landscape are also displayed as the background. Each arrow corresponds to a 10-ns segment of the MD simulation. The black cross corresponds to the holo crystal structure.
(TIF)

Figure S8Fraction of the unbound state as a function of time for the ligand binding to metastable state S3. Eq. 19 is fitted (solid line) to data obtained from MD simulations (points) to derive the kinetic parameters: 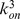 and 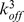.(TIF)

Figure S9 Definition of the twisting and opening dihedral angle. (a) The opening (left panel, side view) and twisting (right panel, top view) angles are defined as angles between pairs of planes. (b) The opening and twisting angles have a good correlation with the top two eigenvectors obtained from the Principal Component Analysis. The correlation coefficients R^2^ are 0.77 and 0.53 between the first eigenvector and the opening angle, and between second eigenvector and the twisting angle, respectively. The protein conformations from all the apo ChoX MD simulations are included in this analysis.
(TIF)

Figure S10 Fractions of conformational selection computed by using two sets of kinetic rates obtained by different definitions of successfully binding events. In the first definition (blue), the distances between the center of mass (c.o.m) of the ligand and the c.o.m of the side-chains of four critical residues in the binding pockets all have to be smaller than 12 Å. In the second definition (red), heavy atoms of the ligand form contact with atoms belonging to at least 3 critical residues in the binding pocket.
(TIF)

Figure S11 Protein conformational changes during the MD simulations in the presence of the ligand (a) S1+L. In particular, the ligand binding occurs in the first 5 panels. Only 9 out of 30 MD simulations of S1+L are displayed. (b) S3+L. The projections of the apo ChoX free energy landscape The projections of the free energy landscape onto the opening and twisting angles are shown for state S1 (Red), S2 (Green) and S3 (Cyan) as background. Each arrow corresponds to a 10-ns segment of the MD simulation. The black cross corresponds to the holo crystal structure.
(TIF)

Figure S12The thermodynamic cycle used for 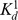 calculation. The ligand dissociation constant *K_d_* is measured from experiments. Δ*G_d_* can also be obtained from a two-step process: protein conformational transition and ligand binding to state S1. Therefore, we can construct a thermodynamic cycle to obtain the value of Δ*G_1_*.(TIF)

Figure S13 Fractions of conformational selection as a function of ligand concentration obtained from the flux analsyis with original rates and with the upper limit of certain rates (see Table 1) are shown in blue and red respectively.
(TIF)

Table S1 Mean first passage times between pairs of macrostates.
(PDF)

Table S2 Rates computed by different definitions of successful ligand binding events. In the first definition, the distances between the center of mass (c.o.m) of the ligand and the c.o.m of the side-chains of four critical residues in the binding pockets all have to be smaller than 12 Å. In the second definition, heavy atoms of the ligand form contact with atoms belonging to at least 3 critical residues in the binding pocket.
(PDF)

Text S1 Force field parameters for choline (ffbonded_choline.itp).
(PDF)

Text S2 Force field parameters for choline (choline.rtp).
(PDF)
