# Supplementary figures and images for: Quantitatively Characterizing the Ligand Binding Mechanisms of Choline Binding Protein Using Markov State Model Analysis
Source: PLoS Comput Biol. 2014 Aug 7;10(8):e1003767. doi: 10.1371/journal.pcbi.1003767 (PMC4125059; doi:10.1371/journal.pcbi.1003767)

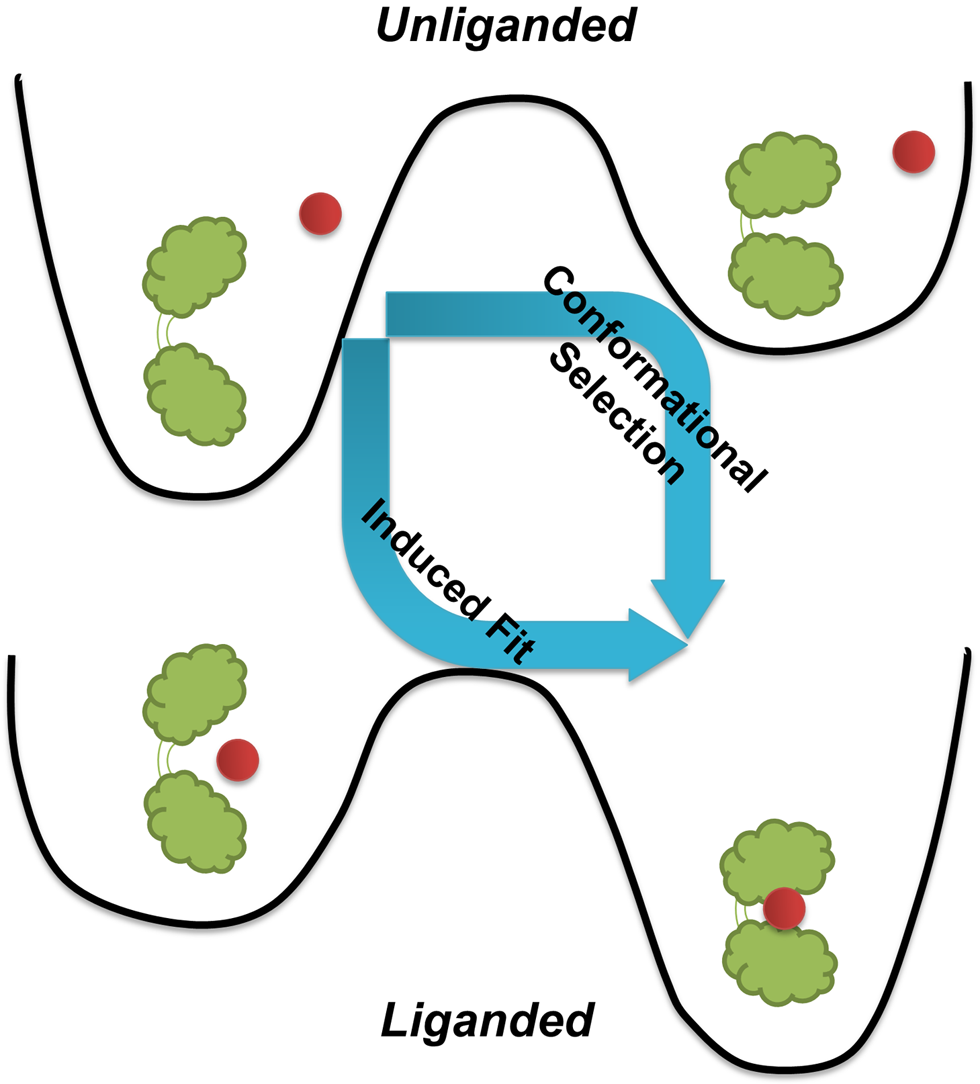

Supplement: Figure S1 — A schematic diagram representing the conformational selection and induced fit models of protein-ligand recognition. (TIF) [file pcbi.1003767.s001.tif]

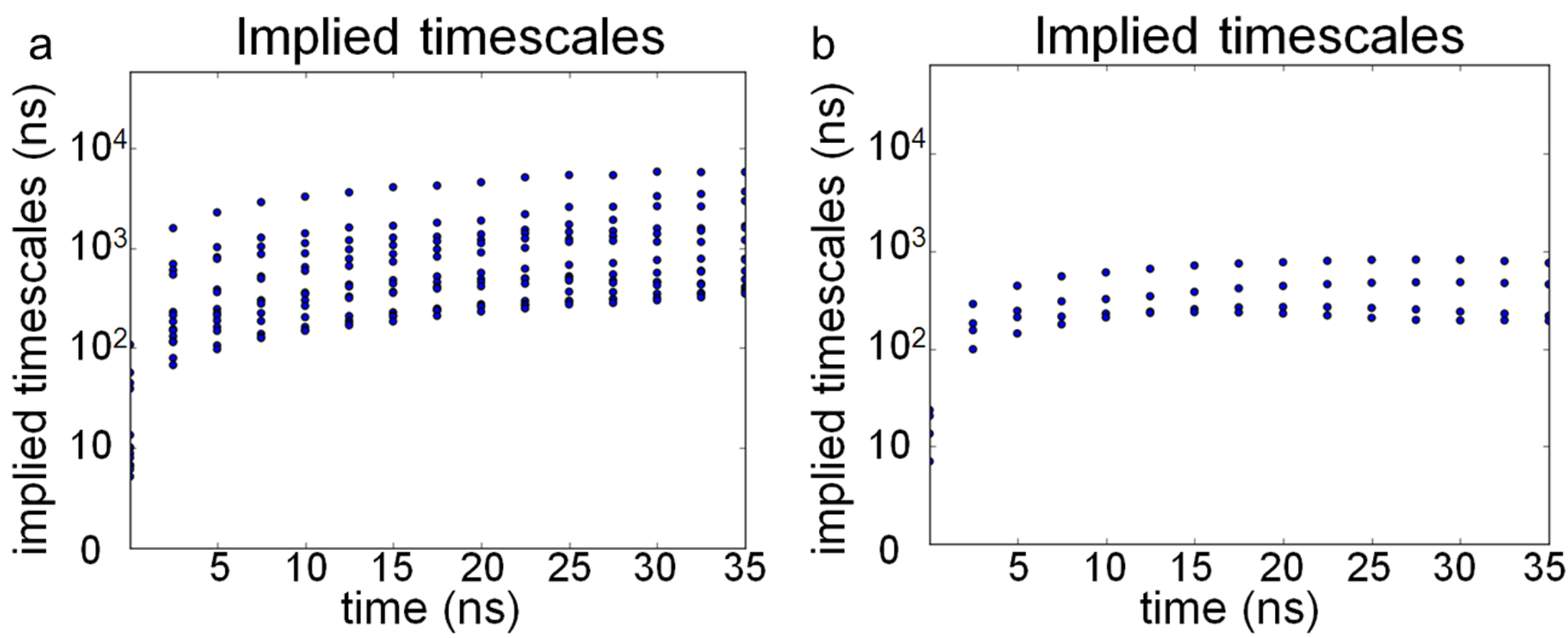

Supplement: Figure S2 — Implied timescales of microstate and macrostate models. (a). Fifteen slowest implied timescales as a function of lag time computed from the 500-state microstate-MSM. (b). Implied timescales as a function of lag time computed from the 5-state macrostate-MSM. (TIF) [file pcbi.1003767.s002.tif]

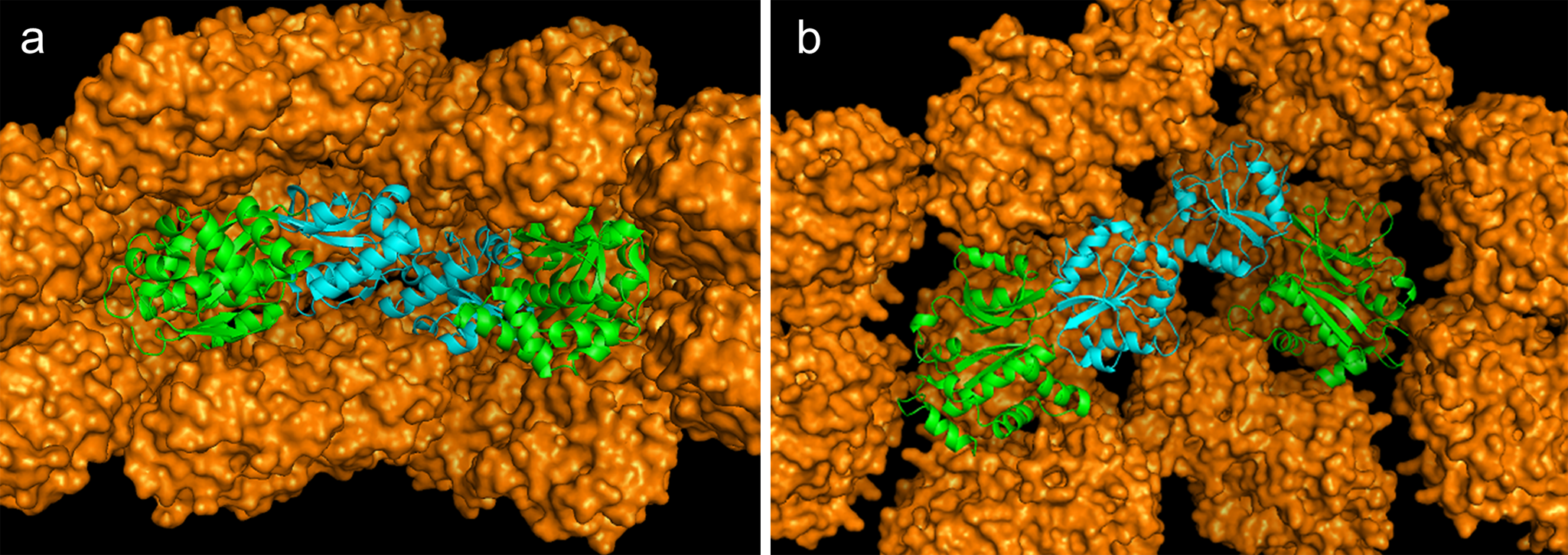

Supplement: Figure S3 — Crystal contacts experienced by the ChoX in the (a) apo-closed (PDB ID: 2RF1) and (b) apo-semiclosed (PDB ID: 2REJ) X-ray structures. The central unit cell contains two ChoX molecules (in ribbon representations). The surrounding unit cells are shown in surface representations in orange. (TIF) [file pcbi.1003767.s003.tif]

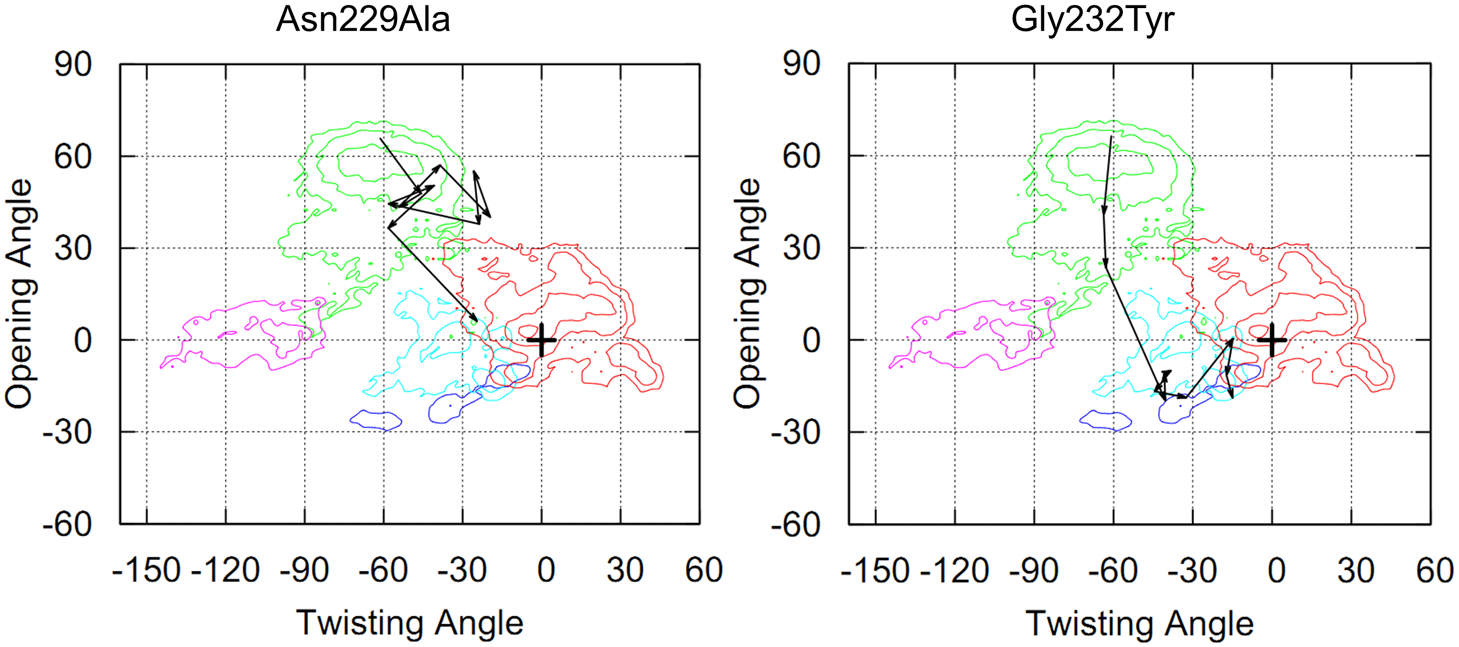

Supplement: Figure S4 — MD simulations of two single mutants of apo ChoX, Asn229Ala and Gly232Tyr, that exhibit an accelerated conformational change from the open to the closed state. We have performed three 50-ns MD simulations for each mutant. One of these MD simulations for each mutant is projected onto the opening and twisting angles with a step size of 5-ns. The projections of the apo ChoX free energy landscape (the same as Fig. 3c, and each macrostate is assigned a different color) are also displayed in the same figure. (TIF) [file pcbi.1003767.s004.tif]

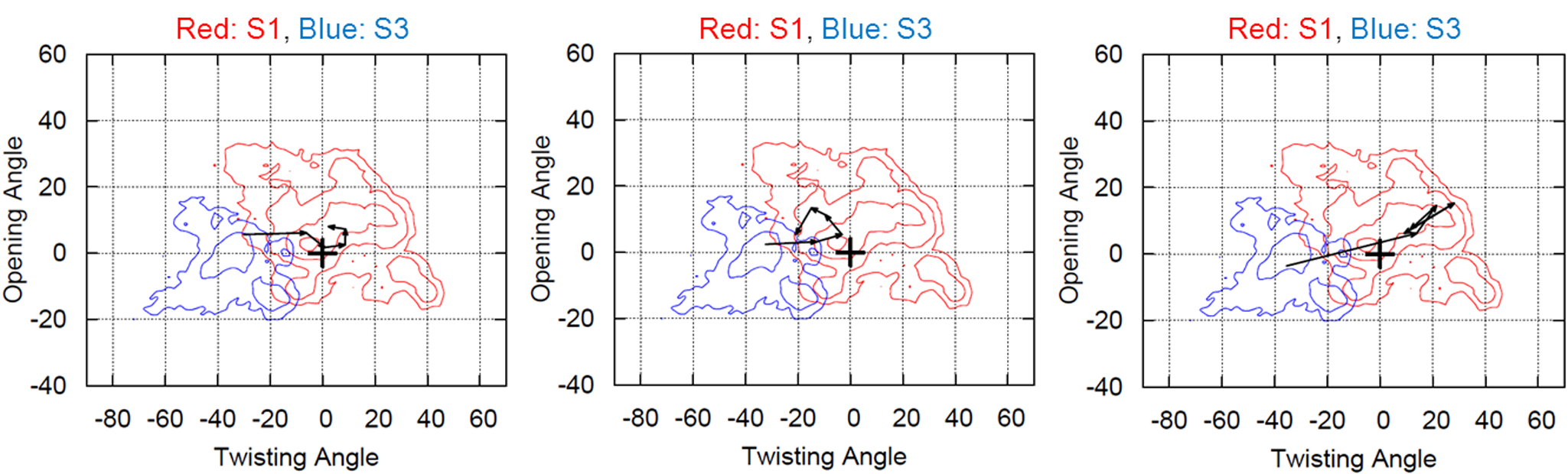

Supplement: Figure S5 — Protein conformational changes are displayed for three MD simulations where transitions from S3L to S1L occur. The projections of the free energy landscape onto the opening and twisting angles are shown for state S3 (blue) and S1 (red). Each arrow corresponds to a 10-ns segment of the MD simulation. The black cross represents the holo crystal structure. The middle and right panels correspond to the two additional MD simulations containing the transitions from S3L to S1L. (TIF) [file pcbi.1003767.s005.tif]

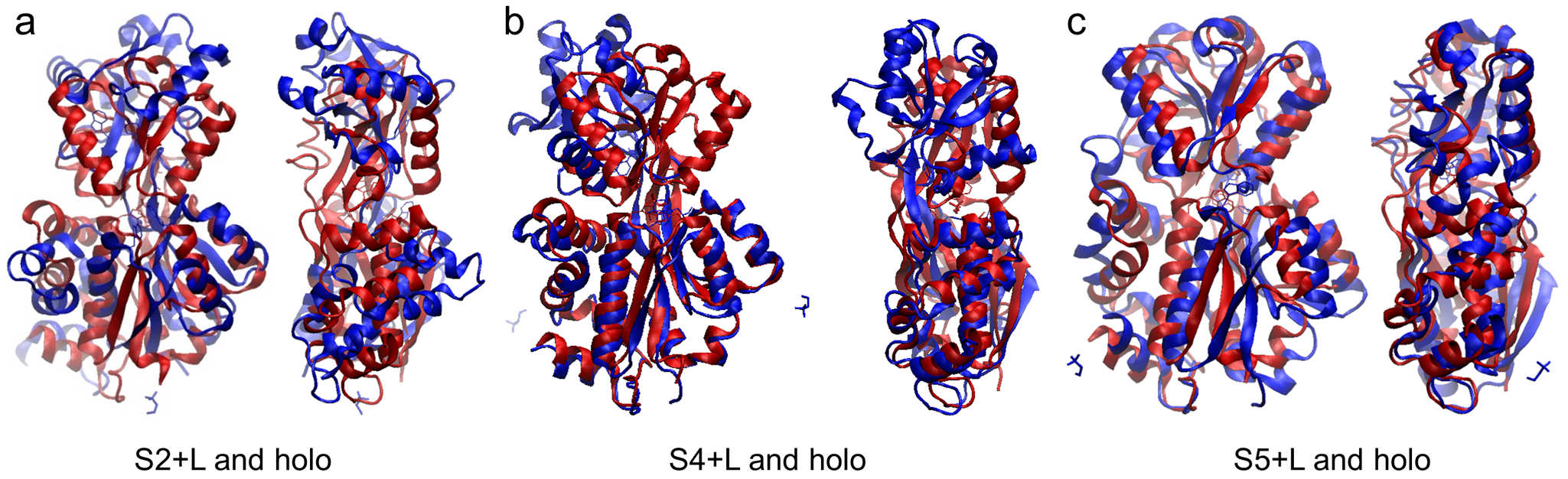

Supplement: Figure S6 — Superimposition of representative snapshots from macrostates S2 (a), S4 (b) and S5 (c) in blue with the X-ray structure of the ChoX bound state (red, PDB ID: 2REG). Each protein conformation is displayed in both front and side views. We did not observe any stable binding events in our MD simulations. A binding event is defined as when distances between the center of mass of the ligand and center of mass of four critical residues (W43, W90, Y119 and W205) in the binding site all to be less than 12 Å. Therefore we consider that the ligands do not bind to these metastable states (S2, S4 and S5). (TIF) [file pcbi.1003767.s006.tif]

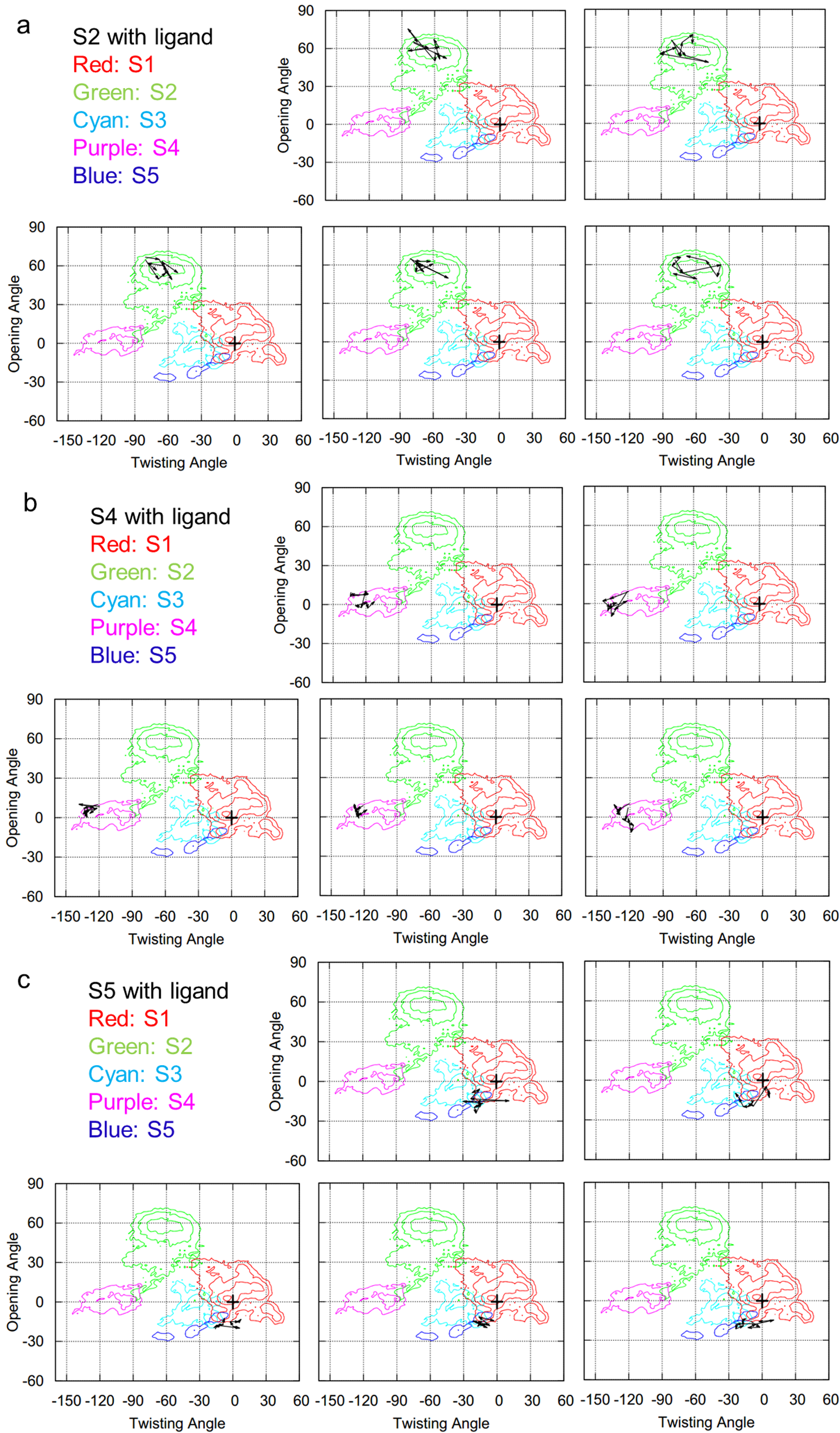

Supplement: Figure S7 — The projections of protein conformational change on the opening and twisting angles during the course of MD simulations are displayed in black arrowed lines (two trajectories in one panel). The simulations initiated from S2L, S4L, and S5L are plotted in (a), (b), and (c) respectively. The projections of the apo protein free energy landscape are also displayed as the background. Each arrow corresponds to a 10-ns segment of the MD simulation. The black cross corresponds to the holo crystal structure. (TIF) [file pcbi.1003767.s007.tif]

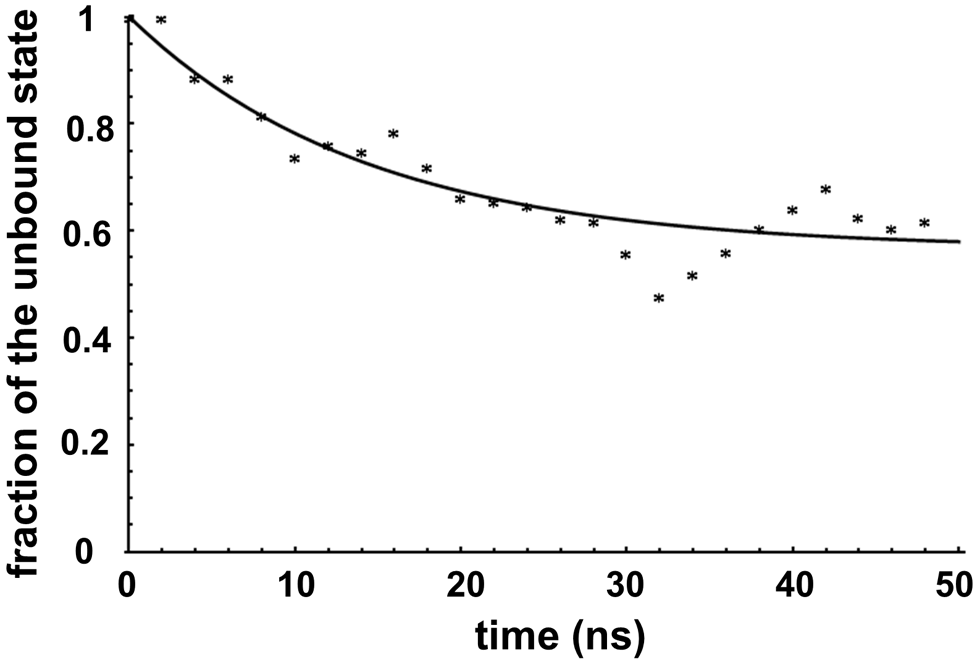

Supplement: Figure S8 — Fraction of the unbound state as a function of time for the ligand binding to metastable state S3. Eq. 19 is fitted (solid line) to data obtained from MD simulations (points) to derive the kinetic parameters: and . (TIF) [file pcbi.1003767.s008.tif]

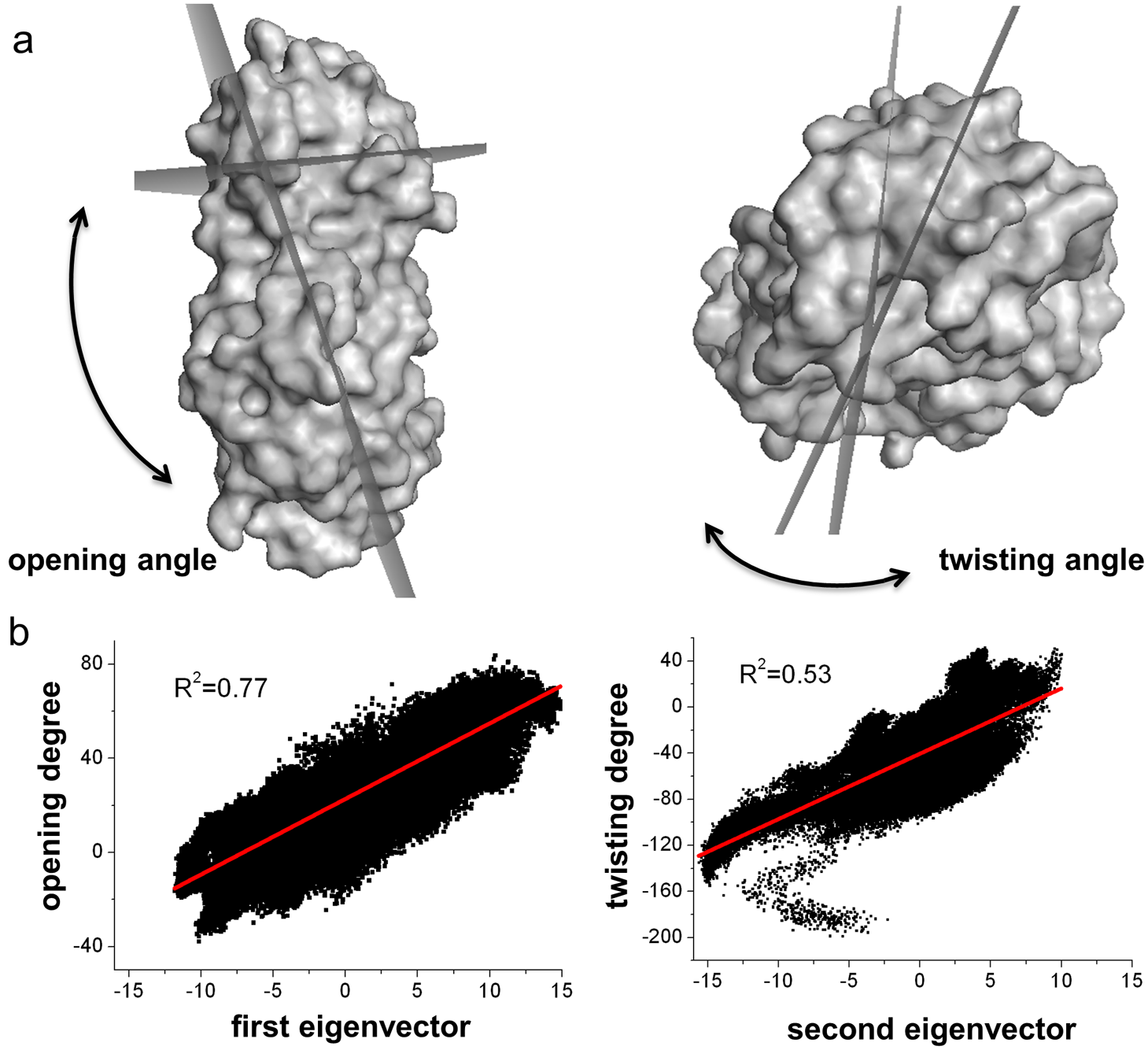

Supplement: Figure S9 — Definition of the twisting and opening dihedral angle. (a) The opening (left panel, side view) and twisting (right panel, top view) angles are defined as angles between pairs of planes. (b) The opening and twisting angles have a good correlation with the top two eigenvectors obtained from the Principal Component Analysis. The correlation coefficients R2 are 0.77 and 0.53 between the first eigenvector and the opening angle, and between second eigenvector and the twisting angle, respectively. The protein conformations from all the apo ChoX MD simulations are included in this analysis. (TIF) [file pcbi.1003767.s009.tif]

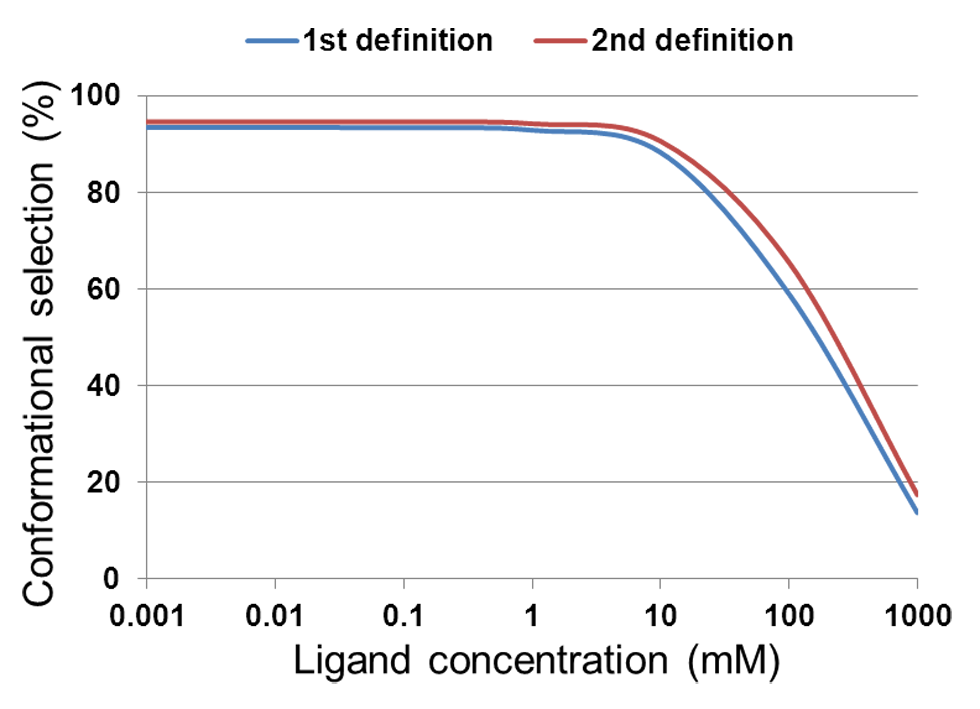

Supplement: Figure S10 — Fractions of conformational selection computed by using two sets of kinetic rates obtained by different definitions of successfully binding events. In the first definition (blue), the distances between the center of mass (c.o.m) of the ligand and the c.o.m of the side-chains of four critical residues in the binding pockets all have to be smaller than 12 Å. In the second definition (red), heavy atoms of the ligand form contact with atoms belonging to at least 3 critical residues in the binding pocket. (TIF) [file pcbi.1003767.s010.tif]

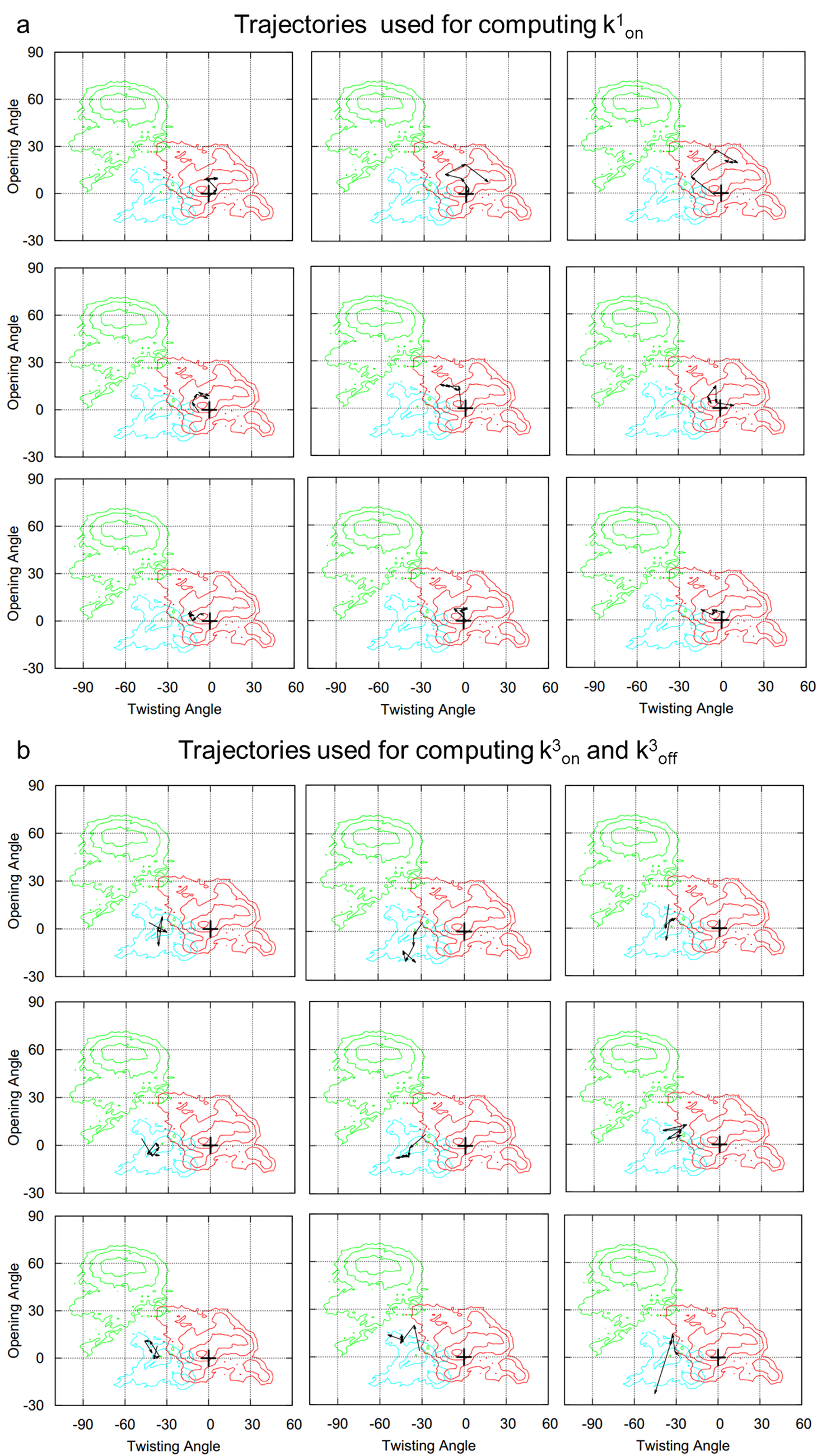

Supplement: Figure S11 — Protein conformational changes during the MD simulations in the presence of the ligand (a) S1+L. In particular, the ligand binding occurs in the first 5 panels. Only 9 out of 30 MD simulations of S1+L are displayed. (b) S3+L. The projections of the apo ChoX free energy landscape The projections of the free energy landscape onto the opening and twisting angles are shown for state S1 (Red), S2 (Green) and S3 (Cyan) as background. Each arrow corresponds to a 10-ns segment of the MD simulation. The black cross corresponds to the holo crystal structure. (TIF) [file pcbi.1003767.s011.tif]

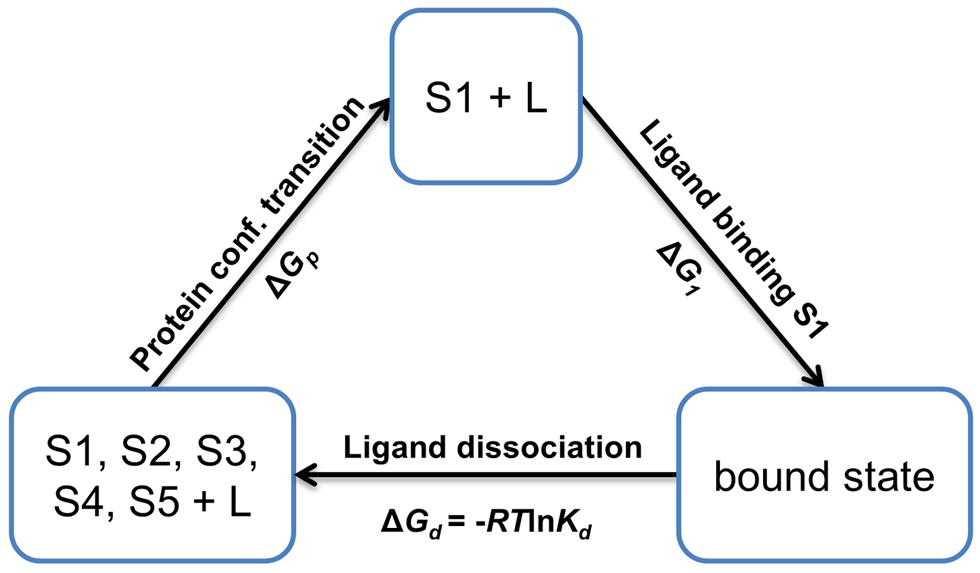

Supplement: Figure S12 — The thermodynamic cycle used for calculation. The ligand dissociation constant Kd is measured from experiments. ΔGd can also be obtained from a two-step process: protein conformational transition and ligand binding to state S1. Therefore, we can construct a thermodynamic cycle to obtain the value of ΔG1. (TIF) [file pcbi.1003767.s012.tif]

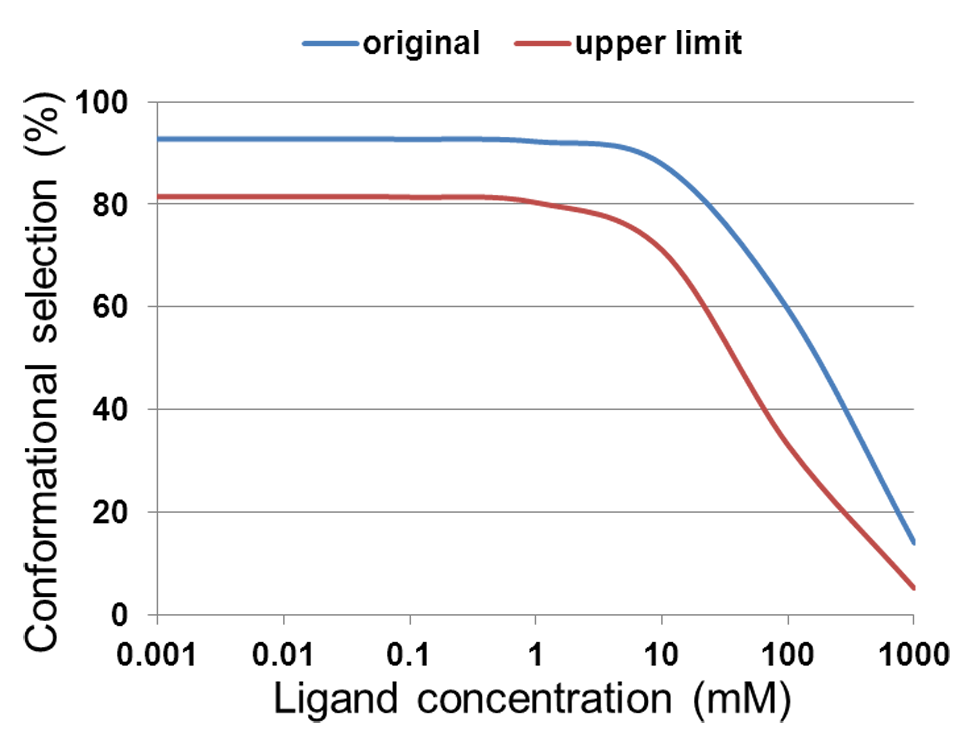

Supplement: Figure S13 — Fractions of conformational selection as a function of ligand concentration obtained from the flux analsyis with original rates and with the upper limit of certain rates (see Table 1) are shown in blue and red respectively. (TIF) [file pcbi.1003767.s013.tif]
