# Supplementary material for: Quantitatively Characterizing the Ligand Binding Mechanisms of Choline Binding Protein Using Markov State Model Analysis
Source: PLoS Comput Biol. 2014 Aug 7;10(8):e1003767. doi: 10.1371/journal.pcbi.1003767 (PMC4125059; doi:10.1371/journal.pcbi.1003767)
Supplement: Table S1 — Mean first passage times between pairs of macrostates. (PDF) [file pcbi.1003767.s014.pdf]

**Table S1.**

| MFPT(μs) | S1 | S2 | S3 | S4 | S5 |
|---|---|---|---|---|---|
| S1 | | 12.08±11.00 | 23.93±17.59 | 35.83±28.28 | 40.40±32.63 |
| S2 | 2.07±1.01 | | 33.02±17.10 | 20.70±20.10 | 35.80±22.49 |
| S3 | 2.71±3.19 | 16.20±9.58 | | 39.95±28.22 | 44.69±31.63 |
| S4 | 9.35±10.01 | 6.15±9.15 | 40.36±20.26 | | 39.13±22.16 |
| S5 | 3.15±4.92 | 7.91±4.36 | 32.92±19.91 | 28.11±20.94 | |
