# Supplementary material for: Quantitatively Characterizing the Ligand Binding Mechanisms of Choline Binding Protein Using Markov State Model Analysis
Source: PLoS Comput Biol. 2014 Aug 7;10(8):e1003767. doi: 10.1371/journal.pcbi.1003767 (PMC4125059; doi:10.1371/journal.pcbi.1003767)
Supplement: Table S2 — Rates computed by different definitions of successful ligand binding events. In the first definition, the distances between the center of mass (c.o.m) of the ligand and the c.o.m of the side-chains of four critical residues in the binding pockets all have to be smaller than 12 Å. In the second definition, heavy atoms of the ligand form contact with atoms belonging to at least 3 critical residues in the binding pocket. (PDF) [file pcbi.1003767.s015.pdf]

Table S2.

	Definition #1		Definition #2
k^1_{on}	6.33×10^7 $M^{-1}s^{-1}$	k^1_{on}	5.88×10^7 $M^{-1}s^{-1}$
k^3_{on}	4.19×10^8 $M^{-1}s^{-1}$	k^3_{on}	3.51×10^8 $M^{-1}s^{-1}$
k^3_{off}	4.50×10^7 s^{-1}	k^3_{off}	5.05×10^7 s^{-1}
