# Supplementary material for: Quantitatively Characterizing the Ligand Binding Mechanisms of Choline Binding Protein Using Markov State Model Analysis
Source: PLoS Comput Biol. 2014 Aug 7;10(8):e1003767. doi: 10.1371/journal.pcbi.1003767 (PMC4125059; doi:10.1371/journal.pcbi.1003767)
Supplement: Text S1 — Force field parameters for choline (ffbonded_choline.itp). (PDF) [file pcbi.1003767.s016.pdf]

Text S1.

```
[ bondtypes ]
; i    j          func     b0         kb
CTL CTL          1      0.15350   253630.0
CTL OHL          1      0.14260   262840.0
CTL H1L          1      0.10930   281080.0
CTL N3L          1      0.14990   245680.0
CTL HPL          1      0.10910   283420.0
OHL HOL          1      0.09740   309280.0

[ angletypes ]
;  i    j    k            func      th0       cth
CTL CTL N3L          1      114.3200   539.3200
CTL CTL HPL          1      111.7400   385.1000
CTL OHL HOL          1      108.1600   394.0500
CTL CTL OHL          1      109.4300   566.6800
CTL CTL H1L          1      110.0700   387.9400
CTL N3L CTL          1      110.6400   525.8500
OHL CTL H1L          1      109.8800   426.5200
N3L CTL HPL          1      107.9100   410.2000
H1L CTL H1L          1      109.5500   327.8600
HPL CTL HPL          1      110.7400   326.6900

[ dihedraltypes ]
;i  j   k  l    func     phase     kd     pn
CTL CTL N3L CTL  9 0.0 1.77466 2
CTL CTL N3L CTL  9 0.0 0.395553 3
CTL CTL OHL HOL  9 180.0 2.32803 1
CTL CTL OHL HOL  9 0.0 2.56256 2
CTL CTL OHL HOL  9 0.0 1.44623 3
CTL N3L CTL HPL  9 0.0 0.898473 3
OHL CTL CTL N3L  9 180.0 4.80502 1
OHL CTL CTL N3L  9 0.0 1.13979  2
OHL CTL CTL N3L  9 0.0 1.65992  3
OHL CTL CTL HPL  9 0.0 0.829962 3
H1L CTL CTL N3L  9 0.0 0.829962 3
H1L CTL CTL HPL  9 0.0 0.141981 3
H1L CTL OHL HOL  9 0.0 0.723115 3
```
