# Supplementary material for: Quantitatively Characterizing the Ligand Binding Mechanisms of Choline Binding Protein Using Markov State Model Analysis
Source: PLoS Comput Biol. 2014 Aug 7;10(8):e1003767. doi: 10.1371/journal.pcbi.1003767 (PMC4125059; doi:10.1371/journal.pcbi.1003767)
Supplement: Text S2 — Force field parameters for choline (choline.rtp). (PDF) [file pcbi.1003767.s017.pdf]

Text S2.

[CHT]

[atoms]

C4	CTL	0.333680	1
C5	CTL	-0.131150	2
N1	N3L	0.102004	3
C6	CTL	-0.405317	4
C7	CTL	-0.405317	5
C8	CTL	-0.405317	6
O6	OHL	-0.695328	7
H41	H1L	0.002700	8
H42	H1L	0.002700	9
H51	HPL	0.155329	10
H52	HPL	0.155329	11
H61	HPL	0.202032	12
H62	HPL	0.202032	13
H63	HPL	0.202032	14
H71	HPL	0.202032	15
H72	HPL	0.202032	16
H73	HPL	0.202032	17
H81	HPL	0.202032	18
H82	HPL	0.202032	19
H83	HPL	0.202032	20
H6	HOL	0.472397	21

[bonds]

C4	C5
C4	O6
C4	H41
C4	H42
C5	N1
C5	H51
C5	H52
N1	C6
N1	C7
N1	C8
C6	H61
C6	H62
C6	H63
C7	H71
C7	H72
C7	H73
C8	H81
C8	H82
C8	H83
O6	H6
